# Comparison of Australian Recommended Food Score (ARFS) and Plasma Carotenoid Concentrations: A Validation Study in Adults

**DOI:** 10.3390/nu9080888

**Published:** 2017-08-17

**Authors:** Lee Ashton, Rebecca Williams, Lisa Wood, Tracy Schumacher, Tracy Burrows, Megan Rollo, Kristine Pezdirc, Robin Callister, Clare Collins

**Affiliations:** 1 Faculty of Health and Medicine, School of Health Sciences, University of Newcastle, Callaghan, NSW 2308, Australia; lee.ashton@newcastle.edu.au (L.A.); rebecca.williams@newcastle.edu.au (R.W.); tracy.schumacher@newcastle.edu.au (T.S.); Tracy.Burrows@newcastle.edu.au (T.B.); Megan.rollo@newcastle.edu.au (M.R.); Kristine.pezdirc@newcastle.edu.au (K.P.); 2 Priority Research Centre in Physical Activity and Nutrition, University of Newcastle, Callaghan, NSW 2308, Australia; Robin.Callister@newcastle.edu.au; 3 Priority Research Centre in Physical Activity and Nutrition, Faculty of Health and Medicine, School of Biomedical Sciences and Pharmacy, University of Newcastle, Callaghan, NSW 2308, Australia; lisa.wood@newcastle.edu.au; 4 Gomeroi gaaynggal Centre, Department of Rural Health, University of Newcastle, Tamworth, NSW 2340, Australia

**Keywords:** validation, dietary methods, diet quality index, food frequency questionnaire, comparative validity

## Abstract

Diet quality indices can predict nutritional adequacy of usual intake, but validity should be determined. The aim was to assess the validity of total and sub-scale score within the Australian Recommended Food Score (ARFS), in relation to fasting plasma carotenoid concentrations. Diet quality and fasting plasma carotenoid concentrations were assessed in 99 overweight and obese adults (49.5% female, aged 44.6 ± 9.9 years) at baseline and after three months (198 paired observations). Associations were assessed using Spearman’s correlation coefficients and regression analysis, and agreement using weighted kappa (K_w_). Small, significantly positive correlations were found between total ARFS and plasma concentrations of total carotenoids (*r* = 0.17, *p <* 0.05), β-cryptoxanthin (*r* = 0.18, *p <* 0.05), β-carotene (*r* = 0.20, *p <* 0.01), and α-carotene (*r* = 0.19, *p <* 0.01). Significant agreement between ARFS categories and plasma carotenoid concentrations was found for total carotenoids (K_w_ 0.12, *p* = 0.02), β-carotene (K_w_ 0.14, *p <* 0.01), and α-carotene (K_w_ 0.13, *p <* 0.01). In fully-adjusted regression models the only signification association with ARFS total score was for α-carotene (β = 0.19, *p <* 0.01), while ARFS meat and fruit sub-scales demonstrated significant relationships with α-carotene, β-carotene, and total carotenoids (*p <* 0.05). The weak associations highlight the issues with self-reporting dietary intakes in overweight and obese populations. Further research is required to evaluate the use of the ARFS in more diverse populations.

## 1. Introduction

Optimal diet quality can be described as alignment of an individual’s usual dietary intake with National Dietary Guidelines and includes the concepts of nutrient intake adequacy and food variety within key healthy food groups [1]. Poor diet quality, characterized by lower intakes of nutrient dense foods and higher intakes of energy-dense, nutrient-poor foods increases the risk of obesity, hypertension, hyperlipidaemia, type 2 diabetes, cardiovascular disease (CVD), and all-cause morbidity and mortality [1,2,3]. While collection of accurate dietary intake and overall diet quality is challenging [4], valid assessment of food and nutrient intakes is important in understanding their relationship with health and the development or prevention of disease. 

Traditional methods of dietary assessment can be burdensome [5]. Prospective methods, including weighed or estimated food records require recording all food and drinks consumed within a defined time period (usually three or seven days). Food records require a high level of literacy and motivation to frequently weigh, measure, or estimate foods [4]. These demands can discourage individuals from adhering to the dietary assessment methods and elicit a conscious or unconscious change in usual eating behaviours (e.g., consume less or choose to eat foods that are easier to prepare and/or report to reduce the burden of recording) [6]. Retrospective methods, including 24 h recalls, can have a substantial burden due to the extensive training required for interviewers and those responsible for data entry including coding of reported food and beverage items, processing, analysis, and quality control [7], although the emergence of free web-based software (i.e., ASA-24) has reduced some of this burden [8]. Food frequency questionnaires (FFQs) enable assessment of longer-term dietary intake in a cost-effective and timely manner [9] with lower respondent burden compared to other methods. However, there are considerable limitations including; reporting errors related to incomplete food lists, voluntary or involuntary misreporting, inaccuracies in frequency options, and portion sizes used [10], and the time they take to complete [4]. 

Another approach is to develop short-form versions of longer FFQ’s, which still capture key aspects of usual dietary intake. Brief dietary assessment tools including diet quality and variety scores or indexes have been developed to provide a single continuous variable that can be used as an overall indicator of nutrient quality and these are increasingly used in research as proxies for assessing overall dietary intakes, due to their low cost and lower subject and analytic burden [11]. These brief tools have the potential to be used as stand-alone questionnaires and may be especially useful in large, nationally representative surveys [11,12]. This is the approach used by a number of a priori diet quality scores [1], including the Australian Recommended Food Score (ARFS) [13] and Healthy Eating Index [14]. Importantly, a review of diet quality tools has shown that they are able to quantify the risk of some health outcomes, including biomarkers of disease and risk of CVD, some cancers, and mortality [1].

There is a need for balance between collection of valid and reliable data versus burden on participants and researchers. The ARFS has previously demonstrated acceptable reproducibility (ICC: 0.87, 95% CI 0.83, 0.90) and validity (0.53, 95% CI 0.37–0.67) in adults compared to the nutrient intakes estimated from the FFQ from which it is derived [13]. However, this approach to validity carries the risk of correlated errors [15]. Plasma biomarkers provide objective assessments of nutrients, and independent measures of intake when validating dietary assessment tools [16]. Therefore, the primary aim of the current study was to examine the relationship between the total ARFS and fasting plasma carotenoid concentrations in a sample of adults. The secondary aim was to examine the associations between the ARFS sub-scales and fasting plasma carotenoid concentrations.

## 2. Materials and Methods 

### 2.1. Participants

Data were obtained from a subset of participants from a previous weight loss RCT (Clinical Trials Registry—ANZCTR, number: ACTRN12610000197033) with methods and primary analysis published in detail elsewhere [17]. Briefly, the population sample included in the current analysis were overweight (BMI 25.0 to 29.9 kg/m^2^) or obese (BMI ≥ 30.0 kg/m^2^) adults, aged 18–60 years, recruited from the Hunter region of New South Wales, Australia from October to December 2009. Assessments were conducted at baseline and three months in the Human Performance Laboratory at the University of Newcastle, Australia, Callaghan campus. Individuals included in the current analysis were those who completed the Australian Eating Survey (AES) from which the ARFS was calculated and provided a blood sample for assessment of plasma carotenoid concentrations at both baseline and after three months’ follow-up. Participants were equally selected from quartiles of baseline fruit and vegetable intake to ensure the data had a spread of low to high intakes. This study was conducted according to the guidelines laid down in the Declaration of Helsinki and all procedures involving human subjects/patients were approved by the University of Newcastle Human Research Ethics Committee (approval No. H-2010-1170). Written informed consent was obtained from all study participants. 

### 2.2. Australian Recommended Food Score 

The ARFS is derived from the AES FFQ [18] and uses a subset of 70 questions related to core nutrient-dense foods recommended in the Australian Dietary Guidelines [19]. The ARFS is calculated by summing the points within eight sub-scales as shown in Table 1, with 20 questions related directly to vegetable intake, 12 to fruit, 13 to protein foods (seven to meat and six to vegetarian sources of protein), 12 to breads/cereals, 10 to dairy foods, one to water, and two to spreads/sauces. The total score ranges from zero to a maximum of 73 points. Briefly, most foods were awarded one point for a reported consumption of ≥once per week, but differed for some items depending on national dietary guideline recommendations [19] with consideration of the Australian Guide to Healthy Eating [20]. Some of the food items for meat (i.e., beef, lamb) and dairy (i.e., ice-cream, frozen yoghurt) had a limit placed on their score for higher intakes due to higher intakes being associated with potentially higher saturated fat or disease risk. Additional points were awarded for greater consumption of vegetables and healthier choices for bread and milk. 

### 2.3. Plasma Carotenoids

Phlebotomists collected blood samples in EDTA-coated tubes after an overnight fast and samples were processed then stored at −80 °C until thawed for analysis. High-performance liquid chromatography was used to determine plasma carotenoid concentrations of α-carotene, β-carotene, lycopene, β-cryptoxanthin, and lutein/zeaxanthin. To isolate the carotenoids from the plasma, ethanol was added to allow deproteination followed by ethyl acetate containing internal standards (canthaxanthin), then vortexed and centrifuged (3000 rpm. for 5 min at 4 °C). The supernatant was collected in separate tubes and stored on ice. This process was repeated three times, adding ethyl acetate twice, then hexane to the pellet. Milli-Q (Milli-Q Advantage A10, Merck Millipore, Melbourne, VIC, Australia) water was then added to the pooled supernatant and the mixture vortexed and centrifuged. The supernatant was decanted and placed on a nitrogen evaporator until completely evaporated. The dried extract was reconstituted in dichloromethane:methanol (1:2 vol/vol). Chromatography was performed on Agilent 1200 series gas chromatograph (Agilent Technologies, Santa Clara, CA, USA, part No. G1311-90011) including Chemstation (Chemstation OpenLab CDS software, Agilent Technologies, Melbourne, VIC, Australia) data analysis software at a flow rate of 0.3 mL/min. Carotenoids were analysed using a mobile phase of acetonitrile:dichloromethane:methanol 0.05% ammonium acetate (85:10:5), and integrated and analysed at a wavelength of 450 nm. Total carotenoids were calculated by summing β-carotene, lycopene, α-carotene, β-cryptoxanthin, and lutein/zeaxanthin concentrations.

### 2.4. Statistical Methods

Data were analysed using Stata Version 12 (StataCorp, College Station, TX, USA) using an alpha level of 0.05. The data from each participant at baseline and 3-months were treated as independent variables. The strength of association between the ARFS or its sub-scales and plasma carotenoid concentrations were assessed in three ways:

1. Spearman’s correlations coefficients were used due to non-normal distribution of plasma carotenoid concentrations. Correlation strength was described as poor <0.20, moderate 0.2–0.6 and strong >0.6 [22].
2. Linear regression models to standardized variables, with standard errors clustered on unique participant identifiers and 95% confidence intervals to examine the relationship between specific plasma carotenoids and component of ARFS, while adjusting for known influential factors including: baseline values for total energy intake, total fat intake, age, sex, and BMI. *R*^2^ values and coefficients (95% CI) are also reported, with *R*^2^ ≥ 0.26 considered large, ≥0.13–<0.26 medium and ≤0.02 small [23].
3. Precision of the agreement of between categorical assessments of ARFS and plasma carotenoid values was tested using weighted kappa (K_w_) statistics to assess whether the ARFS correctly classified participants into the same tertiles of intake based on both total ARFS or ARFS sub-scales and plasma concentrations.

## 3. Results

A total of 99 overweight or obese adults (49.5% female) completed the AES (from which ARFS was calculated) and provided plasma carotenoid concentration assessments at both baseline and three months. The majority were non-smokers (94.9%), with a mean ARFS of 33 ± 9 points from a maximum of 73. Mean energy intake of participants (10,550 kJ/day ± 3581) was comparable to the average Australian adult aged >18 years (9955 kJ/day) [24]; plasma levels of carotenoids were also comparable to the weighted mean of plasma carotenoid concentrations from a systematic review of 142 studies [25] (156 μg/dL vs. 169 μg/dL—converted to micrograms/deciliter for comparison purposes). The highest concentrations were for lutein/zeaxanthin (60 ± 54) and lycopene (44 ± 31). Table 2 summarizes the subject baseline characteristics. 

Table 3 summarizes crude Spearman’s correlations between the total ARFS or individual ARFS sub-scales and the total or individual plasma carotenoid concentrations. The total ARFS diet quality score was significantly associated with plasma concentrations of total carotenoids (*r* = 0.17, *p* < 0.05), β-cryptoxanthin (*r* = 0.18, *p* < 0.05), β-carotene (*r* = 0.20, *p* < 0.01), and α -carotene (*r* = 0.19, *p* < 0.01). Moderate correlations (*r* > 0.20) were found between the ARFS fruit subscale score and α-carotene, β-carotene, and β-cryptoxanthin, and between the combined fruit and vegetable score and plasma concentrations of α-carotene and β-carotene. In addition, moderate correlations were found between the ARFS meat subscale score and plasma concentrations of β-carotene, β-cryptoxanthin and total carotenoids. There were other statistically significant correlations found, however, these were classified as poor correlations (*r* < 0.20). 

Table 4 and Table 5 summarize the unadjusted and the adjusted linear regression analyses between total ARFS and sub-scale component scores and total and individual plasma carotenoid concentrations. In the adjusted regression model (adjusted for total energy intake, total fat intake, BMI, age and sex) total ARFS scores significantly explained the variation in α-carotene (*p* < 0.01), and the ARFS fruit sub-scale significantly explained the variation in α-carotene (*p* < 0.001) and β-carotene (*p* < 0.05), while the ARFS combined fruit and vegetables score explained the variation in α-carotene (*p* < 0.001). The ARFS meat sub-scale explained the variation in plasma concentrations of α-carotene, β-carotene and total carotenoids (all *p* < 0.05).

Table 6 summarizes the analyses examining the extent of agreement between tertiles of ARFS score and tertiles of specific plasma carotenoid concentrations using kappa statistics. Level of agreement indicated by kappa statistics was significant for total ARFS score when compared to plasma concentrations of α-carotene (K_w_ 0.13, *p* < 0.01), β-carotene (K_w_ 0.14, *p* < 0.01), and total carotenoids (K_w_ 0.12, *p* = 0.02). So too was ARFS fruit sub-scale with plasma concentrations of α-carotene (K_w_ 0.16, *p* < 0.01), β-carotene (K_w_ 0.19, *p* < 0.001), β-cryptoxanthin (K_w_ 0.15, *p* < 0.01) and total carotenoids (K_w_ 0.16, *p* < 0.01). Significant agreement was also evident for ARFS combined fruit and vegetable score with α-carotene (K_w_ 0.11, *p* = 0.03) and β-carotene (K_w_ 0.14, *p* < 0.01). There was significant agreement for ARFS meat sub-scale with plasma concentrations of α-carotene (K_w_ 0.11, *p* = 0.02), β-carotene (K_w_ 0.11, *p* = 0.03), β-cryptoxanthin (K_w_ 0.19, *p* < 0.01) and total carotenoids (K_w_ 0.12, *p* = 0.01). Additionally, there was significant agreement for ARFS grains sub-scale with plasma concentrations of lutein/zeaxanthin (K_w_ 0.14, *p* < 0.01), and for ARFS dairy sub-scale with β-cryptoxanthin (K_w_ 0.09, *p* = 0.03). 

## 4. Discussion

The current study examined the relative validity of using a brief diet quality score using the ARFS, in relation to a range of fasting plasma carotenoid concentrations in a sample of overweight and obese adults. The total ARFS was significantly associated with plasma concentrations of total carotenoids, β-carotene and α-carotene as shown by stronger positive correlations, and a small proportion of values misclassified within tertiles of ARFS and plasma carotenoid concentrations. Only the association between total ARFS and α-carotene remained significant in the fully-adjusted regression analyses, which suggests that the ARFS is influenced by factors such as energy intake, fat intake, BMI, sex, and age, which is similar to other dietary variables [27]. However, the ARFS appears to be able to quantify intakes of food sources of α-carotene, such as orange juice, tangerines, raspberries, and tomato sauce-based dishes (e.g., spaghetti bolognaise) [28,29]. This is important because consumption of diets rich in carotenoids have been epidemiologically associated with a lower risk for several diseases, including cancer [30], with the antioxidant properties of carotenoids playing an important role in this. Specifically for α-carotene, research from a prospective cohort study in >15,000 US adults found that people with higher plasma α-carotene concentrations were at a significantly lower risk of death from CVD (*p* = 0.007), cancer (*p* = 0.02), and all other causes (*p* < 0.001) when compared to those with low levels over the 14-year follow-up period [31].

For the individual ARFS sub-scales, both meat and fruit demonstrated significant associations with some of the plasma carotenoid concentrations as shown by; (i) stronger correlations; and (ii) significant associations in the fully-adjusted regression analyses. Specifically, after adjusted regression analysis, fruit was significantly associated with α-carotene and β-carotene, while meat was significantly associated with total carotenoids, β-carotene, and α-carotene. The association of the fruit subscale with plasma concentrations of α-carotene may be attributed to the high content within orange juice which is considered one of the most highly consumed fruits among Australian adults [32]. Association of the ARFS meat sub-scale with plasma carotenoid concentrations may be driven by foods with high β-carotene and α-carotene content, such as vegetables and tomato-based sauces commonly served with meat (i.e., spaghetti bolognaise). The items that form the scoring for the meat subscale are based on questions from the FFQ which asks the type of meat intake (beef, lamb, chicken, or pork) with (or without) vegetables or sauce. This supports previous evidence which has shown that higher intakes of unprocessed red meat, chicken, and fish are associated with higher intakes of vegetables [33]. The significant association of fruit and meat with plasma carotenoids, which remains after adjusting for confounders suggests that a wider range of plasma carotenoids appear to be better predictors of specific food group intake rather than whole diet. This is likely due to food groups, such as spreads/sauces and vegetarian sources of protein which influence total ARFS to weaken associations. 

Although the ARFS vegetable sub-scale demonstrated significant associations with α-carotene and β-carotene using correlation analysis, the significant association no longer remained in the adjusted regression analysis. Plasma concentrations of lutein/zeaxanthin had poor associations with total ARFS and individual subscales of ARFS for all analyses conducted. Lutein/zeaxanthin are most commonly found in green leafy vegetables (e.g., kale, spinach, broccoli, peas, and lettuce) and egg yolks, with the highest content found in kale [34]. There were no questions assessing kale intake in the AES FFQ and thus may explain the poor associations. 

In parallel with similar studies, the strongest correlations between diet and plasma carotenoids were for α-carotene, β-carotene and β-cryptoxanthin [25]. This may be attributed to these carotenoids being highly prevalent in commonly consumed fruit such as apples, apricots, oranges, tangerines, and peaches [29,32]. It is widely acknowledged that individuals are more likely to meet fruit targets than vegetable targets [35]. This may also explain the lack of associations of diet with plasma lutein/zeaxanthin as these are abundant in vegetable sources such as dark greens, including spinach, kale, and broccoli [29]. Limited associations with lycopene may be due to the increased variety of foods containing this carotenoid [29], hence, making it difficult to pick up an association, or due to the lack of foods contributing to lycopene within the ARFS score.

A recent systematic review of 142 biomarker studies comparing plasma carotenoid concentrations and dietary carotenoid intakes, determined mean correlation values by meta-analysis of all relevant studies and gave R values classified as moderate, ranging from 0.26 to 0.47 across the different dietary methods [25]. When diet was assessed using an FFQ, associations appeared to be the weakest compared to other methods and is likely due to the limited range of included food items within FFQ food lists. Additionally, FFQs assess food intake for a long period of time (i.e., six months), while the half-life of plasma carotenoids is 26–76 days [36]. This may explain the weaker associations observed in this current study, as diet quality assessed from ARFS was derived from a subset of FFQ questions. Furthermore, the AFRS does not capture the variation of the AES FFQ as it collapses responses down to two categories. Therefore, some of the contribution to carotenoid intake of those consuming frequently (i.e., multiple times per week) is likely to be lost. Furthermore, the FFQ was not able to determine how food was consumed (i.e., raw, cooked with fat, etc.) which may influence the bioavailability of carotenoids from food. Although correlations in the current study were lower than most of the full-scale dietary measures in the review [25], our results did demonstrate a potential role for use of the ARFS, as some associations were within range (albeit at the lower end of the range) of the full assessment, particularly for fruit. 

### Limitations

Using plasma carotenoids as biomarkers of components of dietary intake has some limitations. For example, plasma carotenoid concentrations can be influenced by a number of dietary, metabolic and lifestyle factors including: the intra and inter variability in individual’s digestion and absorption and the amount of fat in the diet [25]. Despite this, adjustments were made in the regression analyses to account for potential confounders. Although supplements were not adjusted for, previous research using this dataset found no influence on results [37]. For most foods, the scoring for ARFS was based on consumption of greater than, or equal to, once per week. To better quantify diet quality, future studies could include a more comprehensive feature of frequency through more response options. Additionally, the ARFS included spreads/sauces as a sub-scale (namely vegemite as a source of B-group vitamins and tomato or barbecue sauce as a source of or β-carotene), which may have influenced the results as these are generally not considered a component of higher diet quality. However, the overall consumption was low so the effect is deemed to be small. The samples used were from overweight and obese individuals. As carotenoids have an antioxidant role in the body, previous research suggested that people who are overweight or obese have lower plasma concentrations of carotenoids than healthy weight people [38]. Additionally, overweight and obese individuals are more likely to over-report healthy foods such a fruit and vegetables which may influence results [39]. The lack of association between the ARFS vegetable sub-scale and plasma carotenoid concentrations, suggests that this tool may not validly predict vegetable intake and/or variety, likely due to misreporting of vegetable intake. Plasma carotenoid concentrations are considered to be the ideal biological markers of fruit and vegetable intake [40] they are invasive, expensive and responsive to short term intakes [41]. This has implications for measuring a person’s usual or long term intakes. In particular, issues have been raised regarding single biomarker assessment compared to FFQ analysis which report longer term intakes of food [42]. The AES FFQ used in this current study reflects intake over the previous 6 months, but plasma carotenoids have a short half-life and may be more reflective of shorter term intake. Therefore, other dietary assessment methods such as 24 h recall or diet history are likely to have higher associations than a FFQ [25]. However, in the current study observations from two time-points (baseline and three-months) for plasma carotenoid concentrations were used. Responses from the AES FFQ were self-reported and therefore subject to reporting bias [43]. Finally, this study compared the precision of agreement with weighted kappa (K_w_) statistics with data categorized into tertiles. This approach has been implemented in previous diet validation studies [44,45,46] and indicates to what extent the dietary assessment tool is able to rank participants correctly and this reflects agreement at individual level [47]. However, this approach is limited in that the percent agreement can include chance agreement [48]. It is recommended to undertake multiple statistical tests to evaluate validity of dietary intake assessment methods [46].

## 5. Conclusions

Consistent with a recent meta-analysis [25], the current study demonstrated that the diet quality of overweight and obese adults, assessed using the Australian Recommended Food Score (ARFS), has a low correlation with plasma carotenoid concentrations. Stronger relationships were evident between total ARFS and ARFS fruit and meat sub-scales with plasma carotenoids, although associations were weaker after adjustment for confounders. Findings further highlight the implications with self-reporting dietary intakes in overweight and obese populations. Further research is required to evaluate use of the ARFS in clinical practice, epidemiologic research and public health interventions, as a brief continuous measure of diet quality, including in more diverse populations.

## Figures and Tables

**Table 1:** Scoring method for items in the ARFS.

Food Group	Items Giving 1 Point	Items Giving More Than 1 Point	ARFS
Vegetables	3–4 nightly meals with vegetables; ≥1 per week of each of the following vegetables: potato, pumpkin, sweet potato, cauliflower, green beans, spinach, cabbage or Brussels sprouts, peas, broccoli, carrots, zucchini or eggplant or squash, capsicum, corn, mushrooms, tomatoes, lettuce, celery or cucumber, avocado, onion or leek or shallots/spring onion.	2 points for ≥5 nightly meals with vegetables	21
Fruit	≥1 piece of fruit per day, ≥1 per week of each of the following fruit: canned fruit, fruit salad, dried fruit, apple or pear, orange or mandarin or grapefruit, banana, peach or nectarine or plum or apricot, mango or paw-paw, pineapple, grapes or strawberries or blueberries, melon (any variety).		12
Protein foods- meat/flesh	≤1 serve of mincemeat per month but greater than never; 1–4 serve per week of: beef or lamb with or without sauce and/or vegetables per week chicken without batter or crumbing but with or without sauce and/or vegetables, pork with or without sauce and/or vegetables; ≥1 per week of fresh fish, canned tuna or salmon or sardines, other seafood (e.g., prawns, lobster).		7
Vegetarian sources of protein	≥1 per week of the following: nuts (e.g., peanuts, almonds), nut butters, eggs, soybeans or tofu, baked beans, other beans or lentils (e.g., chickpeas, split peas).		6
Breads and cereals (grains)	Usual bread choice is ‘other’ (e.g., rye, high-fiber white); ≥1 per week of the following: muesli, cooked porridge, breakfast cereal (e.g., Weet-bix, Nutri-grain, Cornflakes), bread or pita bread or toast, English muffin or bagel or crumpet, rice, other grains (e.g., couscous, burghul), noodles (e.g., egg noodles, rice noodles), pasta, tacos or burritos or enchiladas, clear soup with rice or noodles.	2 points if usual bread choice is ‘brown’ (multigrain or whole meal).	13
Dairy	≥2 serves of: milk, yoghurt or cheese per day; ≥1 serve per week but ≤1 serves per day of flavoured milk, ice cream, frozen yoghurt; ≥1 serve per week but ≤4 serves per day of cheese, cheese spread or cream cheese; ≥1 serve per week of plain milk, yoghurt (not frozen), cottage cheese or ricotta.	2 points if usual type of milk is reduced fat milk or skim milk, or soy milk	11
Water	≥4 glasses of water (including tap, unflavoured bottled water, and unflavoured mineral water).		1
Spreads/sauces *	≥1 serve per week of: yeast extract spread; tomato or barbecue sauce		2
Total			73

* Yeast extract spread and tomato ketchup/barbecue sauce were included in the score as they contain a large amount of B-group vitamins or β-carotene respectively [21].

**Table 2:** Baseline characteristics of participants (*n* = 99).

Characteristic	Mean ± SD or *n* (%)
Age (years)	44.6 ± 9.9
Female	49 (49.5%)
**Anthropometric**	
Weight (kg)	93.2 ± 14.5
Height (cm)	171.0 ± 8.7
BMI (kg/m^2^)	31.8 ± 3.8
Overweight *	39 (39.4%)
Obese I *	40 (40.4%)
Obese II *	18 (18.2%)
Obese III *	2 (2.0%)
**Smoking status**	
Non-smoker	94 (94.9%)
**ARFS (total possible score)**	
Total Score (73)	33.0 ± 8.8
Vegetables (21)	12.8 ± 4.2
Fruit (12)	4.7 ± 2.9
Protein—Meat (7)	2.7 ± 1.2
Protein—Vegetarian sources (6)	1.7 ± 1.2
Breads/cereals-Grains (13)	5.3 ± 2.0
Dairy (11)	4.3 ± 1.8
Spreads/Sauces (2)	±0.8
**Plasma Carotenoid concentrations (μg/dL)**	
α-Carotene	7.4 ± 6.1
β-Carotene	29.7 ± 22.7
Lycopene	44.0 ± 31.2
Lutein/zeaxanthin	59.6 ± 53.7
β-Cryptoxanthin	10.5 ± 7.6
Total carotenoids	156.0 ± 82.4

* Defined using World Health Organization cut offs [26]: Overweight: 25.0 to 29.99 kg/m^2^, Obese I: 30.0 to 34.99 kg/m^2^, Obese II: 35.0 to 39.99 kg/m^2^, Obese III: ≥40.0 kg/m^2^.

**Table 3:** Spearman rank correlations between total ARFS and sub-scale scores and plasma carotenoid concentrations (based on 198 paired observations).

	α-Carotene	β-Carotene	Lycopene	Lutein/Zeaxanthin	β-Cryptoxanthin	Total Plasma Carotenoids
**Total ARFS**	0.192 **	0.195 **	0.041	0.074	0.180 *	0.170 *
**ARFS—Vegetables**	0.189 **	0.194 *	−0.050	0.002	0.103	0.095
**ARFS—Fruit**	0.278 ***	0.260 ***	0.038	0.047	0.217 **	0.193 **
**ARFS—Fruit and vegetables combined**	0.266 ***	0.258 ***	−0.028	0.027	0.158 *	0.157 *
**ARFS Meat**	0.195 **	0.215 **	0.092	0.002	0.254 ***	**0.209**
**ARFS Vegetarian alternatives**	0.026	0.048	−0.070	0.048	0.007	0.029
**ARFS Grains**	0.012	−0.007	0.098	0.107	0.123	0.072
**ARFS Dairy**	−0.033	−0.015	0.056	0.084	0.082	0.041
**ARFS Spreads/sauces**	0.037	−0.006	0.075	−0.064	−0.022	−0.015

* *P*-value < 0.05; ** *p*-value < 0.01; *** *p*-value < 0.001.

**Table 4:** Unadjusted regression analyses between participant ARFS and plasma carotenoid concentrations (based on 198 paired observations).

Variable	α-Carotene			β-Carotene			Lycopene			Lutein/Zeaxanthin			β-Cryptoxanthin			Total Carotenoids		
	β	95% CI	*R*^2^	β	95% CI	*R*^2^	β	95% CI	*R*^2^	β	95% CI	*R*^2^	β	95% CI	*R*^2^	β	95% CI	*R*^2^
**Total ARFS**	0.19 ***	0.08, 0.30	0.03	0.86 *	0.11, 1.60	0.02	0.31	−0.20, 0.82	0.01	0.08	−0.94, 1.11	0.00	0.07	−0.04, 0.19	0.01	1.51 *	0.06, 2.96	0.02
**ARFS—Vegetables**	0.23	−0.00, 0.46	0.01	1.40	−0.39, 3.18	0.01	−0.01	−1.05, 1.03	0.00	0.51	−1.99, 3.00	0.00	0.06	−0.18, 0.31	0.00	2.19	−1.11, 5.49	0.01
**ARFS—Fruit**	0.87 ***	0.41, 1.34	0.08	3.56 **	0.92, 6.19	0.04	1.05	−0.66, 2.76	0.01	0.46	−2.09, 3.01	0.00	0.28	−0.03, 0.59	0.01	6.22 **	1.61, 10.83	0.03
**ARFS—Fruit and vegetables**	0.32 ***	0.16, 0.48	0.04	1.54 *	0.17, 2.91	0.03	0.25	−0.49, 0.99	0.00	0.36	−1.18, 1.90	0.00	0.10	−0.06, 0.26	0.01	2.57 *	0.35, 4.79	0.02
**ARFS Meat**	1.29 **	0.35, 2.24	0.04	8.99 **	3.34, 14.64	0.06	3.64	−0.15, 7.44	0.01	−0.86	−4.80, 3.09	0.00	0.94	−0.11, 1.77	0.03	14.01 ***	5.57, 22.45	0.04
**ARFS Vegetarian alternatives**	0.54	−0.88, 1.96	0.01	−0.78	−5.73, 4.17	0.00	−2.28	−6.05, 1.48	0.01	1.22	−6.48, 8.92	0.00	−0.30	−1.24, 0.64	0.00	−1.61	−12.56, 9.34	0.00
**ARFS grains**	0.23	−0.35, 0.81	0.00	−0.13	−3.66, 3.41	0.00	1.57	−1.03, 4.17	0.01	−0.19	−5.14, 4.77	0.00	0.24	−0.37, 0.85	0.00	1.72	−5.66, 9.10	0.00
**ARFS Dairy**	−0.05	−0.85, 0.75	0.00	0.04	−4.69, 4.78	0.00	0.99	−3.20, 5.17	0.00	−0.83	−5.11, 3.45	0.00	0.15	−0.50, 0.81	0.00	0.30	−9.40, 10.00	0.00
**ARFS Spreads/sauces**	0.96	−0.72, 2.62	0.00	−1.39	−9.67, 6.89	0.00	5.21	−1.54, 11.97	0.01	−5.16	−14.80, 4.48	0.01	−0.38	−1.75, 1.00	0.00	−0.76	−19.15, 17.63	0.00

β = Regression coefficient. CI = Confidence Interval. *R*^2^ = Partial Correlation coefficient. ARFS = Australian Recommended Food Score. * *p*-value < 0.05; ** *p*-value < 0.01; *** *p*-value < 0.001.

**Table 5:** Adjusted regression analyses between participant ARFS and plasma carotenoid concentrations (based on 198 paired observations).

Variable	α-Carotene			β-Carotene			Lycopene			Lutein/Zeaxanthin			β-Cryptoxanthin			Total Carotenoids		
	β	95% CI	*R*^2^	β	95% CI	*R*^2^	β	95% CI	*R*^2^	β	95% CI	*R*^2^	β	95% CI	*R*^2^	β	95% CI	*R*^2^
**Total ARFS**	0.19 **	0.08, 0.29	0.08	0.43	−0.26, 1.11	0.12	0.38	0.12, 0.88	0.05	0.11	−1.10, 1.32	0.02	0.05	−0.08, 0.17	0.09	1.14	−0.28, 2.57	0.11
**ARFS—Vegetables**	0.20	−0.05, 0.44	0.05	0.73	−0.81, 2.27	0.12	0.09	−0.96, 1.14	0.04	0.51	−2.07, 3.09	0.02	0.01	−0.24, 0.27	0.08	1.54	−1.55, 4.64	0.11
**ARFS—Fruit**	0.90 ***	0.41, 1.39	0.12	2.26 *	0.07, 4.45	0.13	1.41	−0.60, 3.42	0.05	0.50	−2.76, 3.77	0.02	0.13	−0.21, 0.48	0.08	5.21	−0.40, 10.46	0.12
**ARFS—Fruit and vegetables**	0.31 ***	0.15, 0.48	0.08	0.91	−0.25, 2.06	0.12	0.38	−0.41, 1.17	0.04	0.38	−1.34, 2.11	0.02	0.04	−0.14, 0.22	0.08	2.02	−0.19, 4.22	0.12
**ARFS Meat**	1.08 *	0.02, 2.13	0.07	6.03 *	1.50, 10.57	0.14	3.07	−0.63, 6.77	0.05	−2.07	−7.31, 3.18	0.02	0.68	−0.20, 1.56	0.10	8.79 *	0.42, 17.16	0.12
**ARFS Vegetarian alternatives**	0.60	−0.75, 1.94	0.05	−0.64	−5.16, 3.87	0.11	−1.82	−5.56, 1.93	0.04	2.05	−6.25, 10.34	0.02	−0.12	−1.03, 0.80	0.08	0.07	−10.19, 10.33	0.10
**ARFS grains**	0.13	−0.54, 0.80	0.05	−1.85	−6.58, 2.88	0.12	1.49	−1.01, 3.99	0.04	−0.13	−5.54, 5.29	0.02	0.18	−0.39, 0.75	0.08	0.18	−8.54, 8.18	0.10
**ARFS Dairy**	0.13	−0.94, 0.67	0.05	−0.99	−5.46, 3.49	0.12	1.26	−2.69, 5.22	0.04	−0.36	−5.25, 4.53	0.02	0.21	−0.41, 0.84	0.08	−0.00	−9.26, 9.26	0.10
**ARFS Spreads/sauces**	0.91	−0.77, 2.59	0.05	−2.65	−11.22, 5.93	0.12	4.75	−1.68, 11.17	0.05	−5.88	−16.44, 4.69	0.03	−0.21	−1.51, 1.09	0.08	−3.08	−20.89, 14.72	0.10

* Models were adjusted for baseline values for energy intake, total fat intake, BMI, sex, and age. β = regression coefficient. CI = confidence interval. *R*^2^ = partial correlation coefficient. ARFS = Australian Recommended Food Score. * *p*-value < 0.05; ** *p*-value < 0.01; *** *p*-value < 0.001.

**Table 6:** Extent of the agreement between tertiles of ARFS score with tertiles for plasma carotenoids concentrations (based on 198 paired observations).

Variable	*n* = 198 (100%)			Kappa (K_w_)	*p*-Value
	Same Tertile	Adjacent Tertile	Misclassified		
α-carotene					
Total ARFS	80 (40%)	85 (43%)	33 (17%)	0.13	<0.01
ARFS—vegetables	69 (35%)	96 (48%)	33 (17%)	0.07	0.11
ARFS—fruit	79 (40%)	86 (43%)	33 (17%)	0.16	<0.01
ARFS—fruit and vegetables	73 (37%)	93 (47%)	32 (16%)	0.11	0.03
ARFS Meat	70 (35%)	100 (51%)	28 (14%)	0.11	0.02
ARFS Vegetarian alternatives	66 (33%)	88 (44%)	44 (22%)	0.02	0.36
ARFS grains	65 (33%)	78 (39%)	55 (28%)	−0.02	0.62
ARFS Dairy	62 (31%)	99 (50%)	37 (19%)	−0.03	0.72
ARFS Spreads/sauces	65 (33%)	66 (33%)	67 (34%)	−0.01	0.60
β-carotene					
Total ARFS	77 (39%)	90 (45%)	31 (16%)	0.14	0.01
ARFS—vegetables	72 (36%)	93 (47%)	33 (17%)	0.09	0.06
ARFS—fruit	87 (44%)	75 (38%)	36 (18%)	0.19	<0.001
ARFS—fruit and vegetables	78 (39%)	88 (44%)	32 (16%)	0.14	<0.01
ARFS Meat	73 (37%)	95 (48%)	30 (15%)	0.12	0.02
ARFS Vegetarian alternatives	61 (31%)	93 (47%)	34 (17%)	−001	0.54
ARFS grains	58 (29%)	83 (42%)	57 (29%)	−0.06	0.88
ARFS Dairy	79 (40%)	78 (39%)	41 (21%)	0.05	0.13
ARFS Spreads/sauces	66 (33%)	65 (33%)	67 (34%)	−0.01	0.55
Lycopene					
Total ARFS	66 (33%)	92 (46%)	40 (20%)	0.01	0.44
ARFS—vegetables	54 (27%)	99 (50%)	45 (23%)	−0.09	0.95
ARFS—fruit	67 (34%)	85 (43%)	46 (23%)	0.03	0.33
ARFS—fruit and vegetables	61 (31%)	88 (44%)	49 (25%)	−0.06	0.85
ARFS Meat	73 (37%)	91 (46%)	34 (17%)	0.09	0.05
ARFS Vegetarian alternatives	59 (30%)	87 (44%)	52 (26%)	−0.06	0.88
ARFS grains	64 (32%)	85 (43%)	49 (25%)	0.01	0.43
ARFS Dairy	58 (29%)	108 (55%)	32 (16%)	−0.02	0.68
ARFS Spreads/sauces	66 (33%)	67 (34%)	65 (33%)	0.00	0.48
Lutein/Zeaxanthin					
Total ARFS	63 (32%)	100 (51%)	35 (18%)	0.02	0.37
ARFS—vegetables	63 (32%)	97 (49%)	38 (19%)	0.00	0.48
ARFS—fruit	71 (36%)	81 (41%)	46 (23%)	0.05	0.20
ARFS—fruit and vegetables	70 (35%)	86 (43%)	42 (21%)	0.03	0.27
ARFS Meat	59 (30%)	95 (48%)	44 (22%)	−0.05	0.80
ARFS Vegetarian alternatives	70 (35%)	87 (44%)	41 (21%)	0.06	0.14
ARFS grains	78 (39%)	81 (41%)	39 (20%)	0.14	<0.01
ARFS Dairy	63 (32%)	104 (53%)	31 (16%)	0.01	0.39
ARFS Spreads/sauces	59 (30%)	67 (34%)	72 (36%)	−0.07	0.90
β-cryptoxanthin					
Total ARFS	73 (37%)	90 (45%)	35 (18%)	0.08	0.07
ARFS—vegetables	64 (32%)	91 (46%)	43 (22%)	−0.02	0.61
ARFS—fruit	81 (41%)	79 (40%)	38 (19%)	0.15	<0.01
ARFS—fruit and vegetables	68 (34%)	92 (46%)	38 (19%)	0.05	0.19
ARFS Meat	85 (43%)	83 (42%)	30 (15%)	0.19	<0.01
ARFS Vegetarian alternatives	69 (35%)	79 (40%)	50 (25%)	0.01	0.46
ARFS grains	66 (33%)	83 (42%)	49 (25%)	0.02	0.34
ARFS Dairy	77 (39%)	88 (44%)	33 (17%)	0.09	0.03
ARFS Spreads/sauces	64 (32%)	65 (33%)	69 (35%)	−0.03	0.69
Total carotenoids					
Total ARFS	76 (38%)	91 (46%)	31 (16%)	0.12	0.02
ARFS—vegetables	73 (37%)	88 (44%)	37 (19%)	0.06	0.11
ARFS—fruit	75 (38%)	86 (43%)	37 (19%)	0.12	0.02
ARFS—fruit and vegetables	67 (34%)	97 (49%)	34 (17%)	0.06	0.13
ARFS Meat	78 (39%)	86 (43%)	34 (18%)	0.12	0.01
ARFS Vegetarian alternatives	70 (35%)	86 (43%)	42 (21%)	0.06	0.15
ARFS grains	68 (34%)	82 (41%)	48 (24%)	0.04	0.23
ARFS Dairy	66 (33%)	99 (50%)	33 (17%)	0.02	0.31
ARFS Spreads/sauces	60 (30%)	66 (33%)	72 (36%)	−0.06	0.87
